# Counterion Effect and Isostructurality in a Series of Ag(I) Complexes Containing a Flexible, Imidazole Based Dipodal Ligand

**DOI:** 10.3390/ma14071804

**Published:** 2021-04-06

**Authors:** Liliana Dobrzańska

**Affiliations:** Faculty of Chemistry, Nicolaus Copernicus University in Toruń, Gagarina 7, 87-100 Toruń, Poland; lianger@umk.pl

**Keywords:** SCXRD, isostructurality, coordination polymers

## Abstract

The crystal structures of a series of Ag(I) complexes with 1,3-bis(imidazol-1-ylmethyl)-5-methylbenzene (**L**) and the counterions BF_4_^−^ (**1**), PF_6_^−^ (**2**), ClO_4_^−^ (**3**), and CF_3_SO_3_^−^ (**4**) were analysed to determine the effect of the latter on their formation. All resulting compounds crystallise in the non-centrosymmetric space group *Cc* of a monoclinic system and show the formation of cationic, polymeric 1D Ag(I) complexes. SCXRD analyses revealed that compounds **1**–**3** are isostructural, though **1** shows opposite handedness compared to **2** and **3**, resulting in an inversed packing arrangement. The presence of the larger, elongated triflate counterion in **4** leads to a different ligand conformation, as well as different arrangements of the ligand in the cationic chain, and simultaneously results in a packing that exhibits fewer similarities with the remaining three compounds.

## 1. Introduction

The definition of isostructurality provided by the International Union of Crystallography (IUCr) states that ‘Two crystals are said to be isostructural if they have the same structure, but not necessarily the same cell dimensions nor the same chemical composition, and with a ‘comparable’ variability in the atomic coordinates to that of the cell dimensions and chemical composition’ [1].

The phenomenon is of high interest, especially for crystal engineers and the pharmaceutical industry, as it provides valuable information for crystal structure predictions. Many particular cases have been reported up till now [2,3,4,5,6,7]. Notably, a systematic study on the subject was initiated in the nineties of the last century by the Kálmán group. They tried to clarify the existing terminology and to determine the borders of isostructurality by introducing quantitative descriptors, such as a unit-cell similarity index and isostructurality indices [8,9], which resulted in the conception of the program ISOS [10,11]. Furthermore, the search for similarity of molecular arrangements on different levels (0–3 D dimensions) in diverse types of crystals led to the extension of the phenomenon of isostructurality to polymorphs [12,13,14,15]. To facilitate the identification of equal ‘supramolecular constructs’ and the quantification of their similarity, the program X-PAC was developed in 2005 [16].

**Figure 1:**
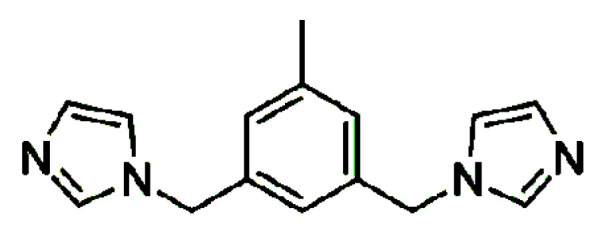
1,3-Bis(imidazol-1-ylmethyl)-5-methylbenzene (**L**).

In continuation of our work concerning metal complexes formed with imidazole based extended ligands [17,18,19], we recently reported the isostructurality of two polymeric 1D Ag(I) complexes containing the CF_3_SO_3_^−^ counterion with two related ligands differing by the presence of a methyl group on the aromatic core, namely 1,3-bis(imidazol-1-ylmethyl)benzene and 1,3-bis(imidazol-1-ylmethyl)-5-methylbenzene (the latter has been further investigated here) [20]. In that report, we suggested that isostructurality is more likely to be achieved by polymeric coordination compounds of particular built, containing ligands differing by small structural modifications, than in the case of their parent ligands (discrete molecules). Following up on this work, two new Ag(I) complexes with 1,3-bis(imidazol-1-ylmethyl)-5-methylbenzene (**L**, Figure 1) and BF_4_^−^ and PF_6_^−^ were prepared and compared with the earlier reported Ag(I)**L** complexes containing the counterions ClO_4_^−^ [21] and CF_3_SO_3_^−^ [20], in order to check the effect of the counterions on the crystal structure formation. One more study on an Ag(I) complex with NO_3_^−^ has been reported, but as the compound is a dihydrate and the water molecules obviously affect the crystal structure formed, it will not be further discussed here [22].

## 2. Materials and Methods

### 2.1. Reagents and Materials

All commercially available chemicals were of reagent grade and were used without further purification. The ligand 1,3-bis(imidazol-1-ylmethyl)-5-methylbenzene (**L**), was synthesized by the S_N_2 reaction of imidazole with 1,3-bis(bromomethyl)-5-methylbenzene in MeOH as reported earlier [20]. A solution of a particular silver salt (0.1 mmol) in acetonitrile (10 mL) was added to a solution of 1,3-bis(imidazol-1-ylmethyl)-5-methylbenzene **L** (0.1 mmol) in acetonitrile (30 mL). The mixture was stirred for a few minutes and then left in dark to undergo slow evaporation. After 3–4 weeks, colourless crystals were obtained. 

### 2.2. Structure Determination

The single-crystal X-ray diffraction data for **1** and **2** were collected on an SuperNova diffractometer (Oxford Diffraction) equipped with an Eos CCD detector and MoKa radiation, λ = 0.71073 Å. The crystals were mounted on a glass fibre and coated with Paratone-N oil. Data collection was carried out at 100(2) K to minimize solvent loss, possible structural disorder and thermal motion effects. Data frames were processed (unit cell determination, intensity data integration, correction for Lorentz and polarisation effects, and empirical absorption correction) by using the corresponding diffractometer’s software package (*CrysAlisPro Software System*, version 1.171.39.46; Rigaku, Oxford, UK) [23]. The structures were solved by using direct methods with SHELXS-2018/3 [24] and refined by using full-matrix least-squares methods based on F2 by using SHELXL-2018/3 [25]. The programs Mercury [26] and POV-Ray [27] were both used to prepare molecular graphics. All non-hydrogen atoms were refined anisotropically, and the hydrogen atoms were positioned geometrically with C-H = 0.95 Å (aromatic), C-H = 0.98 Å (methyl) and 0.99 Å (methylene) and refined as riding, with Uiso(H) = 1.2 Ueq (C) and 1.5 Ueq (methyl C). 

A summary of the data collection and structure refinement parameters is provided in Table 1. For comparison, the unit cell parameters for **3** and **4** are as follows: a = 13.430(11)/13.3761(7) Å, b = 15.280(13)/16.6393(10) Å, c = 8.508(8)/8.5931(5) Å, *β* = 106.48(6)/93.452(3)°, V = 1674(3)/1909.09(19) Å^3^ [20,21].

## 3. Results

### Crystal Structures of Complexes **1**–**4**: {[Ag**L**]counterion}_n_

**Figure 2:**
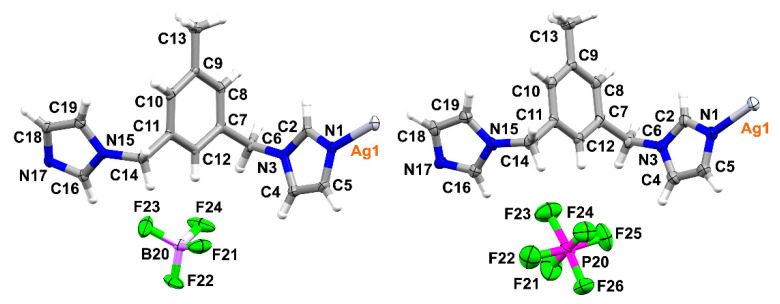
Representation of the asymmetric unit in **1** (**left**) and **2** (**right**); atomic displacement plots are shown with 50% probability.

SCXRD analysis showed that all complexes crystallise in the same space group *Cc* of a monoclinic system with similar unit cell parameters (the biggest deviation is observed for the *b* parameter in **4**). It also revealed the presence of one ligand molecule coordinated with one silver ion, as well as one counterion balancing the positive charge of the silver(I) complex in the asymmetric units of these complexes (Figure 2). 

**Figure 3:**
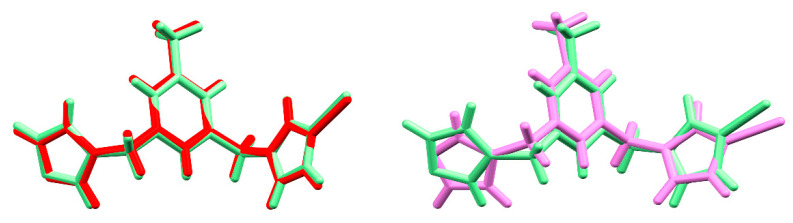
Overlays of silver (I) complexes **1** (green) and **2** (red) on the **left** (RMS deviation is 0.122 Å), and silver (I) complexes **1** (green) and **4** (pink) on the **right** (deviation is 0.939 Å).

The formed cationic complexes show a polymeric composition consisting of a 1D chain with the silver ions linearly coordinated by two N-atoms, originating from two symmetry-related ligands. The 1D chains are running along the *a* axis in the case of **1–3** and are parallel to the [201] direction in the case of **4**. The biggest deviation from linearity in N-Ag-N angle is observed for **4**, with a corresponding value of 175.5(4)°, and could result from weak interactions of the metal centre with the O-atom originating from the counterion (Ag---O distance of 2.81 Å). The corresponding values of N-Ag-N angles for the remaining complexes are 179.6(1)°, 176.9(3)° and 179.3(3)° for **1**, **2** and **3** respectively. The Ag-N bond lengths remain in the range 2.089(7) Å—2.117(6) Å and do not deviate significantly from the corresponding values in related compounds [20,28]. The ligand adopts the *anti* conformation in all four complexes with a dihedral angle between the planes defined by benzene and both imidazole rings of 84.1(1)°/69.7(1)°, 83.1(2)°/72.5(2)°, 82.5°/69.3° [9], 76.3(3)°/70.6(7)° respectively for complexes **1–4**. The dihedral angles between the planes defined by the imidazole rings are equal to 36.4(1)°(**1**), 35.2(4)°(**2**), 35.8°(**3**) [21] and 49.0(8)° for **4**. There is evidently a significant difference in these values between compounds **1–3** and **4**, indicating some conformational alteration in the latter compound. Closer inspection of this structure uncovers that one of the imidazole rings is flipped, and therefore both imidazole rings are pointing up in **4** (with respect to the methyl substituent on the benzene ring) whereas they are alternating in **1–3** (Figure 3).

The cationic Ag(I) chains in **1–4** form undulated layers expanding in the *ac* plane, which are held together by C-H···π interactions. This involves one of the methylene groups of the ligand and a benzene ring (for **1**, also the imidazole ring) originating from an adjacent chain in the case of **1–3**, (Table 2) and both methylene groups of the ligands interacting with all three aromatic rings in **4**. The closest distance between silver ions originating from two adjacent chains in the same layer are 4.7 Å in **1**, 4.9 Å in **2,** 4.7 Å in **3** and 4.4 Å in **4**, respectively. The counterions are located between these layers and interact through an extended net of C-H···F (**1** and **2**), C-H···O (**3**) or a combination of both (**4**) weak hydrogen bonding with all aromatic rings, methyl and methylene groups of the ligands forming 3D supramolecular assemblies (Table 2 and [20], presenting hydrogen bonding geometry details for **4**). 

**Figure 4:**
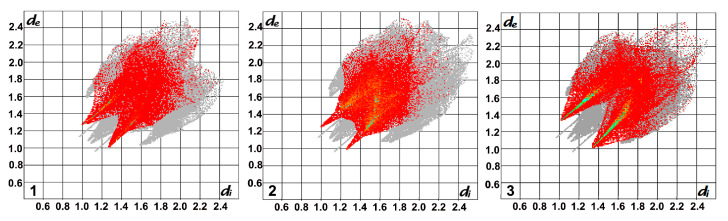
Fingerprint plots of the isostructural complexes **1–3** with the contribution of H···F/F···H (**1**, **2)** and H···O/O···H (**3**) contacts highlighted in red (grey shadows represent the full fingerprints).

The involvement of particular interactions stabilising the crystal structures was estimated by Crystal Explorer [32] a program enabling the visualisation of the molecular packing in a 3D surface (Hirshfeld surface), which can be transposed to a 2D representation of intermolecular distances. These so called fingerprints plot the distances going from the Hirshfeld surface to the closest atoms inside and outside the surface, which are marked as d_e_ and d_i_, respectively [33]. Though these fingerprint plots are unique for each molecule, those for **1–3** are similar in shape as the compounds show isostructurality (Figure 4). The contribution of H···F/F···H (**1–2**) and H···O/O···H (**3**) intermolecular contacts establishing the formation of weak hydrogen bonds stabilising these structures, was calculated as 25.4%, 30.2% and 34.0% for **1–3**, respectively (Figure 4). The higher percentage of former contacts in the case of **2** compared to **1** is not surprising as the compound contains more F atoms. The C-H···F interactions present in **2** involve all available H atoms from both imidazole rings, whereas in case of **1**, the H atoms from C4 and C5 are not involved (see Table 2). Furthermore, H···H type contacts are also frequently encountered in these structures, contributing 32.4%, 26.7% and 29.4% respectively.

**Figure 5:**
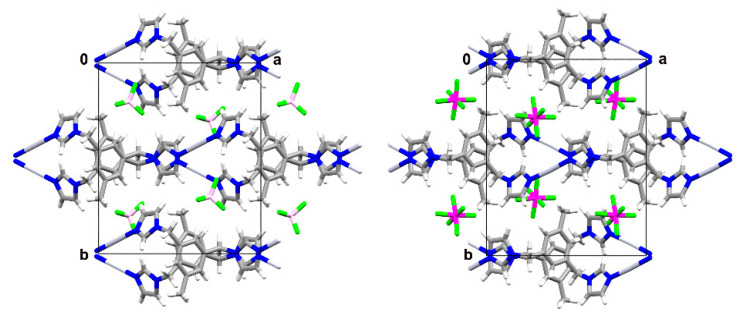
Schematic representation of the crystal packing in **1** (**left**) and **2** (**right**), shown along the *c* axis.

With respect to the unit cells parameters of **1–3**, the biggest discrepancy can be observed for the pair **1** and **2**, with an elongation of the *b* parameter of ca. 0.6 Å in **2**. This is caused by the presence of counterions of different volume (38 Å^3^ for BF_4_^−^ (**1**), 54 Å^3^ for PF_6_^−^ (**2**), 47 Å^3^ for ClO_4_^−^ (**3**), [34]) and the geometry of these anions, facilitating different types of interactions. The cell similarity index (П) in this case is equal to 0.03 and the isostructurality index I(20) = 59.4%. However, these values (in particular the latter) are not fully representative as they are influenced by the big difference in size of the counterions [35]. The cell similarity indices for the pairs **1** and **3** and **2** and **3**, with a smaller, almost equal difference in size between the counterions, show П values of ca. 0.01 and I(20) = 84.5%/79.8%, respectively. The slightly higher value of the isostructurality index for the pair **1–3** versus **2–3** might arise from the same geometry of the counterions in the former case. The most striking difference in molecular arrangement of the compounds **1** and **2/3** is caused by the opposite handedness of the isolated crystals, leading to equal but inversed packing motives (Figure 5). Therefore, it might be more appropriate to refer to this phenomenon as inversed isostructurality.

**Figure 6:**
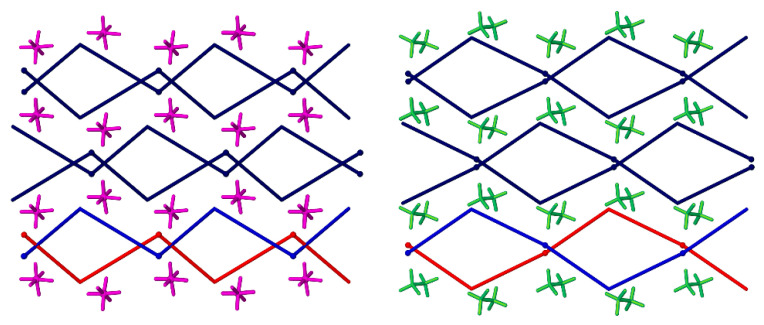
Simplification of the crystal packing in **2** (**left**) and **4** (**right**) shown down the *c* axis, indicating the positions of the silver atoms and the carbon atom of the methyl group on the benzene ring, in the bottom layer, two selected 1D chains are presented in red and blue respectively to indicate the difference, silver atoms shown as balls.

The CF_3_SO_3_^−^ ion present in **4**, has an elongated shape which differs from the remaining counterions, as well as a much bigger anion volume (77 Å^3^) [36], which influence the formation of the crystal structure. Furthermore, it is equipped with O and F atoms, both capable of forming weak hydrogen bonds. As mentioned above, its presence causes a change in the conformation of the ligand. Moreover, a closer look at the formed 1D chain reveals that the monomeric complex units alternating in the chain are related by an inversion centre, causing a different orientation of the methylene group in the mesitylene ring compared to **1–3**. This leads to the presence of more free space between the layers allowing a bigger-sized counterion to fit in (Figure 6), which results in an elongation of the *b* axis of ca. 1.6 Å in comparison with **1** (the closest distances between adjacent chains located in neighbouring layers are ca. 3.6 Å in **1** and **2**, 3.7 Å in **3** and 4.4 Å in **4**), as well as a lower packing efficiency with Kitaigorodskii packing indices of 67.7% in **4** versus 72.8%, 71.2% and 70.9% in **1–3**, respectively.

**Figure 7:**
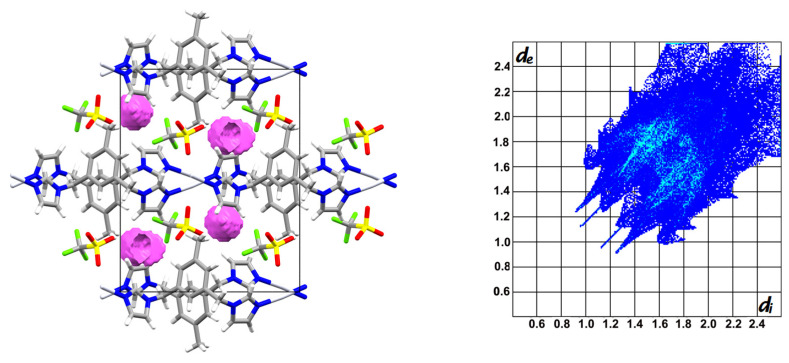
Schematic representation of the crystal packing in **4** shown down the *c* axis, with free space indicated in pink (**left**); fingerprint plot for **4** (**right**).

The latter is a consequence of the irregular shape of CF_3_SO_3_^−^ which excludes fully efficient molecular packing and leads to the presence of free space. Mapping with the application PLATON [37] indicates a volume of 46.9 Å^3^ per unit cell, accounting for 2.5% of the total cell volume (grid = 0.2 Å, probe radius 1.2 Å), which is not available in complexes **1–3** (Figure 7). Furthermore, the differences between **4** and **1–3** are reflected in a lower cell similarity index oscillating around 0.1 between **4** and each of the remaining compounds, as well as a different shape of the fingerprint plot (Figure 7). The latter indicates the stabilisation of the structure by a different set of intermolecular interactions, with H···F/F···H and H···O/O···H contacts contributing more or less equally for a total of 34% (the same input was observed for H···O/O···H contacts in **3**), whereas the contribution of H···H contacts with a percentage of 21.5% is lower than in compounds **1–3**. It is worth mentioning that the larger volume of the trifluoromethanesulfonate anion was reported to preclude ion exchange with perchlorate or hexafluoridophosphate ions [38]. Interestingly, even though **4** is not isostructural with **1–3**, it shows isostructurality with an Ag(I) complex of an analogous ligand without methyl substituent on the benzene ring, namely 1,3-bis(imidazol-1-ylmethyl)benzene. In this case, the volume of the voids between the formed layers was calculated as 124.6 Å^3^ per unit cell, which could indicate that even the lack of a methyl group situated on the ligand‘s benzene ring is not sufficient to gain enough free space to fit CF_3_SO_3_ between the layers formed by the polymeric chains, as is the arrangement in **1–3**.

## 4. Conclusions

Comparison of the crystal structures of a series of Ag(I) complexes formed with 1,3-bis(imidazol-1-ylmethyl)-5-methylbenzene and a range of counterions (BF_4_^−^, PF_6_^−^, ClO_4_^−^ and CF_3_SO_3_^−^; in molar ratio 1:1) revealed that the first three anions lead to isostructurality, with the highest structural similarity indices observed for compounds containing counterions of similar size and geometry, in particular BF_4_^−^ and ClO_4_^−^. SCXRD analyses of the complex containing CF_3_SO_3_^−^, a counterion of much larger size and different shape, revealed that it crystallises in the same space group with similar unit cell parameters as the other three complexes. However, after deeper structural analysis, the initial impression of its isostructurality with the remaining complexes had to be revised, as it indicated changes already on the molecular level, namely a different conformation of the ligand caused by the flip of one of the imidazole rings. Together with the difference in distribution of the monomeric units in the polymeric cationic chain compared to **1–3**, this facilitates the presence of more free space between the formed supramolecular layers to accommodate the larger, elongated counterion. Interestingly, this compound is isostructural with an analogue formed with a ligand without a methyl group on the benzene ring, namely {[Ag(1,3-bis(imidazol-1-ylmethyl)benzene]CF_3_SO_3_}_n_. Further studies on additional representatives of this family of complexes and their packing preferences are ongoing.

## Figures and Tables

**Table 1:** Crystal data and details of the refinement parameters for the crystal structures **1** and **2**.

Parameter	Compound Reference	
	1	2
Chemical formula	C_15_H_16_AgN_4_·BF_4_	C_15_H_16_AgN_4_·PF_6_
Formula Mass	447.00	505.16
Crystal system	Monoclinic	Monoclinic
*a*/Å	13.3836(7)	13.2476(13)
*b*/Å	14.9566(8)	15.5277(10)
*c*/Å	8.3713(5)	8.8493(10)
*α*/°	90	90
*β*/°	106.836(6)	105.538(9)
*γ*/°	90	90
Unit cell volume/Å^3^	1603.88(16)	1753.8(3)
Temperature/K	100(2)	100(2)
Space group	*Cc*	*Cc*
No. of formula units per unit cell, *Z*	4	4
Radiation type	Mo Kα	Mo Kα
Absorption coefficient, *μ*/mm^−1^	1.305	1.309
No. of reflections measured	11179	3791
No. of independent reflections	3787	2557
*R_int_*	0.0313	0.0252
Final *R_1_^a^* values (*I* > 2*σ*(*I*))	0.0252	0.0378
Final *wR_2_^b^* values (*I* > 2*σ*(*I*))	0.0541	0.0773
Final *R_1_^a^* values (all data)	0.0273	0.0492
Final *wR_2_^b^* values (all data)	0.0554	0.0820
Goodness of fit on *F*^2^ Flack parameter CCDC number	1.053 −0.02(1) 2065080	1.033 −0.05(3) 2065081

^a^ R_1_ = ∑║Fo| − |Fc║/∑|Fo|. ^b^ *w*R_2_ = {∑[w(F_o_^2^ − F_c_^2^)_2_]/∑[w(F_o_^2^)_2_]}^1/2^.

**Table 2:** Hydrogen bonding parameters for complexes **1–3**.

Compound	D-H···A	H···A/Å	D···A/Å	D-H···A/°
1	C8-H8···Cg^i^ (imidazole: 1–5)	2.98	3.887(5)	160
	C14-H14B···Cg^ii^ (benzene)	2.82	3.449(5)	122
	C12-H12···F21	2.47	3.420(4)	176
	C18-H18···F21^iii^	2.42	3.324(5)	159
	C2-H2···F22^iv^	2.42	3.142(5)	132
	C6-H6B···F22^v^	2.51	3.306(5)	161
	C13-H13B···F23^iv^	2.72	3.554(6)	143
	C19-H19···F23^vi^	2.87	3.754(6)	156
	C6-H6A···F24	2.48	3.442(6)	164
	C13-H13C···F24^ii^	2.38	3.327(5)	161
	C10-H10···F24^vi^	2.76	3.493(6)	135
2	C14-H14B···Cg^i^ (benzene)	2.96	3.507(10)	116
	C2-H2···F21^ii^	2.81	3.742(10)	165
	C13-H13B···F21^ii^	2.78	3.665(12)	151
	C10-H10···F23^i^	2.37	3.194(11)	145
	C14-H14B···F23^iii^	2.90	3.627(11)	131
	C19-H19···F23^iii^	2.83	3.608(11)	141
	C12-H12···F24	2.51	3.462(8)	177
	C13-H13A···F24^i^	2.75	3.709(11)	167
	C18-H18···F24^iv^	2.71	3.653(10)	170
	C13-H13C···F25^iii^	2.99	3.896(11)	154
	C6-H6B···F26^v^	2.86	3.485(11)	122
3	C11-H5···Cg^i^ (benzene)	2.89	3.499(8)	120
	C12-H6···O1^ii^	2.76	3.568(10)	143
	C51-H15···O1^iii^	2.72	3.588(11)	148
	C14-H8···O1^iv^	2.90	3.785(10)	156
	C2-H1···O2^ii^	2.56	3.506(10)	178
	C13-H7···O2^v^	2.49	3.410(10)	164
	C51---H14···O2^vi^	2.86	3.837(11)	174
	C31-H9···O3^ii^	2.54	3.512(9)	167
	C6-H3···O3^iv^	2.88	3.590(10)	132
	C51-H16···O3^iv^	2.46	3.396(10)	159
	C32-H11···O4^iii^	2.47	3.200(9)	134
	C31-H10···O4^vii^	2.63	3.408(10)	135

Symmetry codes: (**1**): (i) x,-y,−1/2 + z (ii) x,-y,1/2 + z (iii) 1/2 + x,−1/2 + y,z (iv) −1/2 + x,−1/2 + y,z (v) −1/2 + x,1/2-y,−1/2 + z, (vi) x,-y,1/2 + z; (**2**): (i) x,-y,−1/2 + z, (ii) 1/2 + x, −1/2 + y,z, (iii) x,-y,−1/2 + z, (iv) −1/2 + x,−1/2 + y,z, (v) 1/2 + x,1/2-y, 1/2 + z; (**3**) (i) x,-y,−1/2 + z, (ii) 1/2 + x,1/2-y,1/2 + z, (iii) 1 + x,-y,1/2 + z, (iv) 1/2 + x,−1/2 + y,z, (v) x, -y, ½ + z, (vi) 1/2 + x,−1/2 + y,1 + z; (vii) 1 + x,y,1 + z. The cut-off for hydrogen bonding was based on IUPAC recommendations and a book by Desiraju and Steiner [29,30,31].

## Data Availability

Data is contained within the article.

## References

[1] IUCr. http://reference.iucr.org/dictionary/Isostructural_crystals.

[2] Mondal P.K., Shukla R., Biswas S., Chopra D. (2018). Role of halogen-involved intermolecular interactions and existence of isostructurality in the crystal packing of—CF3 and halogen (Cl or Br or I) substituted benzamides. Acta Crystallogr..

[3] Dey D., Chopra D. (2017). Occurrence of 3D isostructurality in fluorinated phenyl benzamidines. CrystEngComm.

[4] Mazur L., Koziol A.E., Jarzembska K.N., Paprocka R., Modzelewska-Banachiewicz B. (2017). Polymorphism and isostructurality of the series of 3-(4,5-diaryl-4H-1,2,4-triazole-3-yl)propenoic acid derivatives. Cryst. Growth Des..

[5] Suresh K., Khandavilli U.B.R., Gunnam A., Nangia A. (2017). Polymorphism, isostructurality and physicochemical properties of glibenclamide salts. CrystEngComm.

[6] Sridhar B., Nanubolu J.B., Ravikumar K., Karthik B., Reddy B.V.S. (2017). Three isostructural solvates of a tetrahydrofurochromenone derivative. Acta Crystallogr..

[7] Czylkowska A., Pietrzak A., Szczesio M., Rogalewicz B., Wojciechowski J. (2020). Crystal structures, Hirshfeld surfaces, and thermal study of isostructural polymeric ladders of La(III) and Sm(III) coordination compounds with 4,4’-Bipyridine and Dibromoacetates. Materials.

[8] Kálmán A., Párkányi L., Argay G. (1993). Classification of the Isostructurality of Organic Molecules in the Crystalline State. Acta Crystallogr..

[9] Fábián L., Kálmán A. (1999). Volumetric measure of isostructurality. Acta Crystallogr..

[10] Kálmán A., Párkányi L., Hargittai M., Hargittai I. (1997). Isostructurality of Organic Crystals in Advances in Molecular Structure Research.

[11] Kálmán A., Gans W. (1996). In Fundamental Principles of Molecular Modeling.

[12] Fábián L., Kálmán A. (2004). Isostructurality in one and two dimensions: Isostructurality of polymorphs. Acta Crystallogr..

[13] Coles S.J., Threlfall T.L., Tizzard G.J. (2014). The Same but Different: Isostructural Polymorphs and the Case of 3-Chloromandelic Acid. Cryst. Growth Des..

[14] Deya D., Thomas S.P., Spackman M.A., Chopra D. (2016). Quasi-isostructural polymorphism in molecular crystals:Inputs from interaction hierarchy and energyframeworks. Chem. Commun..

[15] Jha K.K., Dutta S., Kumar V., Munshi P. (2016). Isostructural Polymorphs: Qualitative Insights from Energy Frameworks. CrystEngComm.

[16] Gelbrich T., Hursthouse M.B. (2005). A versatile procedure for the identification, description and quantification of structural similarity in molecular crystals. CrystEngComm.

[17] Alen J., Van Meervelt L., Dehaen W., Dobrzańska L. (2015). Solvent diffusion through a non-porous crystal ‘caught in the act’ and related single-crystal-to-single-crystal transformations in a cationic dinuclear Ag(I) complex. CrystEngComm.

[18] Dobrzańska L. (2015). Concomitant, genuine 1D supramolecular isomers of an Ag(I) complex with 1,3-bis(imidazol-1-ylmethyl)-2,4,6-trimethylbenzene and BF4^−^ as counterion. Inorg. Chem. Commun..

[19] Dobrzańska L. (2011). Anion directed supramolecular architectures of silver(I) complexes with 1,3-bis(imidazol-1-ylmethyl)-2,4,6- trimethylbenzene and a reversible, solvent-induced structural change during a single-crystal-to-single-crystal transformation. CrystEngComm.

[20] Arhangelskis M., Van Meervelt L., Dobrzańska L. (2021). Influence of ligand composition on crystal structure formation—Isostructurality and morphotropism. CrystEngComm.

[21] Sui B., Fana J., Okamura T., Sun W.-Y., Ueyama N. (2005). Synthesis, structure and properties of Mn(II), Zn(II), Ag(I) and Cu(II) complexes with 1,3-bis(imidazole-1-ylmethyl)-5-methylbenzene. Solid State Sci..

[22] Ma Y., Huang W., Yao J., Li B., Gou S., Fun H.-K. (2003). A three-dimensional zinc(II) interpenetrating network and a π–π induced silver(I) zigzag complex connected by 1,3-di(imidazole-1-yl-methyl)-5-methylbenzene. J. Mol. Str..

[23] Rigaku Oxford Diffraction (2018). CrysAlisPro Software System.

[24] Sheldrick G.M. (2008). A short history of SHELX. Acta Crystallogr..

[25] Sheldrick G.M. (2015). Crystal structure refinement with SHELXL. Acta Crystallogr..

[26] Macrae C.F., Bruno I.J., Chisholm J.A., Edgington P.R., McCabe P., Pidcock E., Rodriguez-Monge L., Taylor R., van de Streek J., Wood P.A. (2008). New Features for the Visualization and Investigation of Crystal Structures. J. Appl. Crystallogr..

[27] Available online: www.povray.org

[28] Fan J., Sun W.-Y., Okamura T., Zheng Y.-Q., Sui B., Tang W.-X., Ueyama N. (2004). Novel Metal-Organic Frameworks with Specific Topology Formed through Noncovalent Br···Br Interactions in the Solid State. Cryst. Growth Des..

[29] Arunan E., Desiraju G.R., Klein R.A., Sadlej J., Scheiner S., Alkorta I., Clary D.C., Crabtree R.H., Dannenberg J.J., Hobza P. (2011). Definition of the hydrogen bond (IUPAC Recommendations 2011). Pure Appl. Chem..

[30] Arunan E., Desiraju G.R., Klein R.A., Sadlej J., Scheiner S., Alkorta I., Clary D.C., Crabtree R.H., Dannenberg J.J., Hobza P. (2011). Defining the hydrogen bond: An account (IUPAC Technical Report). Pure Appl. Chem..

[31] Desiraju G.R., Steiner T. (2006). The Weak Hydrogen Bond in Structural Chemistry and Biology.

[32] Wolff S.K., Grimwood D.J., McKinnon J.J., Turner M.J., Jayatilaka D., Spackman M.A. (2012). CrystalExplorer (Version 3.1).

[33] Spackman M.A., McKinnon J.J. (2002). Fingerprinting intermolecular interactions in molecular crystals. CrystEngComm.

[34] Mingos D.M.P., Rohl A.L. (1991). Size and Shape Characteristics of Inorganic Molecules and Ions and their Relevance to Molecular Packing Problems. J. Chem. Soc. Dalton Trans..

[35] Wood P.A., Oliveira M.A., Zink A., Hickey M.B. (2012). Isostructurality in pharmaceutical salts: How often and how similar?. CrystEngComm.

[36] Georgiadou D.G., Palilis L.C., Vasilopoulou M., Pistolis G., Dimotikali D., Argitis P. (2013). Influence of the anion on the optoelectronic characteristics of triphenylsulfonium salts modified polymer light emitting devices. Synth. Met..

[37] Spek A.L. (2009). Structure validation in chemical crystallography. Acta Crystallogr..

[38] Phuengphai P., Massera C., Reedijk J., Youngme S., Gamez P. (2013). Anion Exchange in Coordination-Network Materials. Eur. J. Inorg. Chem..

